# Injection therapy for prostatic disease: A renaissance concept

**DOI:** 10.4103/0970-1591.42613

**Published:** 2008

**Authors:** Arash M. Saemi, Jeffrey B. Folsom, Mark K. Plante

**Affiliations:** Division of Urology, Department of Surgery, University of Vermont, Burlington, Vermont, USA

**Keywords:** Benign prostatic hyperplasia, injection, prostatic disease, prostate

## Abstract

**Purpose:**

Initially conceived as an intervention for prostatic infection, injection therapy has been used to alleviate urinary retention, and is now primarily investigated for the treatment of lower urinary tract symptoms (LUTS) secondary to benign prostatic hyperplasia (BPH). For over a century, intraprostatic injection has been used as a minimally invasive surgical therapy (MIST), and is on the verge of a rebirth. This review will familiarize the reader with the origins and history of intraprostatic injection, and its evolution using transperineal, transrectal and transurethral routes with multiple injectants.

**Materials and Methods:**

A MEDLINE review of the literature on intraprostatic injections published between 1966 and 2007 was performed, augmented with articles and documents dating back to 1832.

**Results:**

Transperineal and transurethral injections have the most systematic evaluation in patients. There are advantages and disadvantages associated with each route. Most injectants consistently produce localized coagulative necrosis and gland volume reduction with varying degrees of LUTS relief. Anhydrous ethanol (AE) is the most extensively studied injected agent to date.

**Conclusions:**

Injection therapy is a promising minimally invasive treatment option for various prostatic conditions and has been examined for over 100 years. Further experience in systematic laboratory research and completion of currently ongoing clinical trials is necessary before widespread clinical application.

## INTRODUCTION

Injection therapy has a rich and cyclic history in the urologist's armamentarium for treatment of prostatic disease. Initially intended for use as an intervention for prostatitis, its application evolved to ameliorate urinary retention in men. More recently, injection therapy has been primarily investigated for relief of lower urinary tract symptoms (LUTS), with significant attention to its most common causative pathology, benign prostatic hyperplasia (BPH). Over the last century, treatment of LUTS suggestive of bladder outlet obstruction and more progressive disease relating to BPH has largely consisted of extirpative surgery. In recent years, however, technological improvements have seen a paradigm shift from invasive surgical procedures to less invasive techniques with lower associated morbidity. Major efforts are underway toward developing and improving both medical and minimally invasive treatment options.

Despite pharmacological management, significant numbers of patients require definitive surgical intervention for symptomatic disease. Patients who respond poorly to medical treatment, who are averse to transurethral resection of the prostate (TURP) - the current gold standard of surgical treatment - or who are at high risk, may undergo one of numerous alternative treatments made available within the last 15 years. Despite the development of numerous minimally invasive surgical treatments (MIST), most are rapidly abandoned as they are shown to be ineffective, lack reproducible results and have unacceptably high levels of morbidity.[1]

Among the first concepts addressed historically to minimize the morbidity associated with traditional surgical prostate gland volume reduction was injection therapy. References to the first intraprostatic injection date back to more than a century ago. As a promising minimally invasive treatment option that is safe, simple, effective and inexpensive, prostatic injection is on the verge of a rebirth.

We examine and interpret numerous articles relating to intraprostatic injection for the treatment of prostatic disease, with specific attention to BPH. The literature demonstrates many investigative efforts using both animal models and patients to evaluate routes and agents for injection of the prostate. This review examines the historical experience of this concept and addresses, after more than 100 years from its initial inception, why intraprostatic injection is again being considered as a possible minimally invasive solution for the treatment of BPH-related LUTS.

## OVERVIEW

The issue of the shortest and most direct surgical route to the prostate has claimed the attention of urologists for more than a century. In a 1936 manuscript on intraprostatic injections Townsend referenced one of the earliest documented experiences using a needle intraprostatically;[2] Sir B.C. Brodie, in 1832, recommended the treatment of prostatic abscess via a transperineal puncture of the prostate.[3] Three decades later, Hamilton,[4] Maunder[5] and Picard[6] introduced and popularized the transrectal route of prostatic abscess puncture. Picard had the foresight and vision. In an 1896 essay, he stated that the prostatic abscess was accessible through the urethra, rectum or perineum, routes still commonly used today.[7] In 1877 Stoll punctured the prostate through the perineum using a curved trocar which he left *in situ*, allowing subsequent drainage of pus and urine through the hollow cannula.[8] Townsend believed that because the trocar is but a magnified needle, credit was due to Stoll for performing the first intraprostatic injection in 1877.[2] In 1894 Hoffman reported having “had good results in a number of cases” using transperineal and transrectal intraprostatic injections of an antiseptic solution in patients with prostatic abscesses.[9] From 1910 to 1930, antiseptic injectables in the prostate were popularized in the literature as treatments for various forms of prostatic infections, including gonococcal prostatitis.[10,11]

During this same period, intraprostatic injection was being used as a minimally invasive technique by Sir James Roberts, the English surgeon to Lord Hardinge, then Viceroy of India (1909-1916). He used a mixture composed of carbolic acid, distilled water, glacial acetic acid and glycerin to reduce prostatic enlargement and alleviate the associated obstructive symptoms via prostatic injection.[12,13] In 1930, Lower and Johnston postulated that ablation of prostatic tissue could be achieved by injection and made the astute observation that prostatic enlargement seldom followed acute glandular infection, whereby scar tissue replaced the secretory portions of the gland. As such, “it occurred to [them] that [they] might find some chemical agent wherewith a non-infectious prostatitis could be induced with resultant shrinkage of the gland”.[14] Using various chemical agents that included 95% alcohol, Lugol's solution, 5-10% silver nitrate, and 10% sodium hydroxide, they performed transperineal intraprostatic injections on murine and canine models with notable results. Lower and Johnston reported glandular reduction by way of “fibroblastic replacement.” However, only half of the animals survived longer than two weeks.[14,15]

In 1936 Payr reported using the proteolytic enzyme pepsin combined with a 1% iodine solution (Pregl's solution) via transperineal injection of 97 human cases.[15,16] Of the 82 cases of “true hypertrophy” good results were obtained in 72%, and poor results in 28%.

In 1966 Talwar and Pande published the first systematic scientific human evaluation of the injection technique using results from 188 consecutive patients treated for urinary retention.[12] All patients were treated using a solution of carbolic acid, glacial acetic acid, and glycerin via transperineal intraprostatic injection. Many of their study participants were designated high-risk/poor operative candidates with preexisting co-morbidities. The two investigators reported substantial improvement and favorable outcomes in their patients using the injection technique as compared to the traditional retropubic prostatectomy used at the time. In their published work, the authors deduced eight conclusions detailing the advantages of injection therapy that remain applicable to the evolution of this concept and its current trends.[12,17] Talwar and Pande popularized intraprostatic injection.

**Figure 1:**
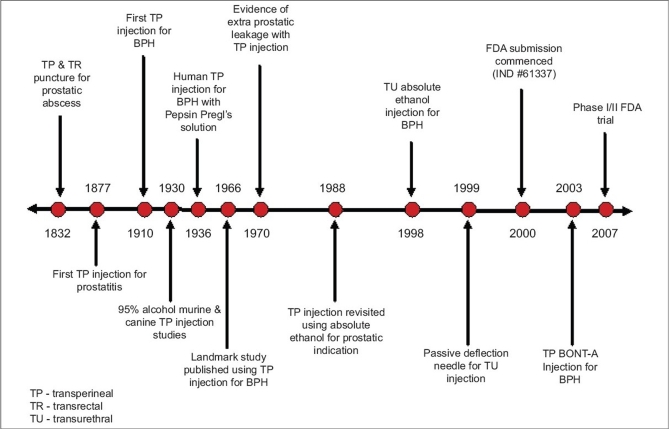
Chronology of Intraprostatic Injection *TP: Transperineal; TU: Transurethral; BPH: Benign prostatic hyperplasia; FDA: Food and Drug Administration; IND: Investigational new drug*

Over the next 40 years, intraprostatic injection became the avant-garde issue for investigators, reducing prostatic volume by necrosing, solubilizing, and eliminating prostate tissue. Differences in the techniques created varied with the route of injection, choice of injectable agent, and patient indication [Table 1]. The transperineal, transrectal and transurethral routes have all been explored in the treatment of BPH. Many injectants have been examined, with the majority reported to induce some degree of inflammatory host response with eventual localized coagulative necrosis, subsequent gland volume reduction, and restoration of varying degrees of LUTS relief. As the routes and agents for injection have historically undergone significant change, so have the indications for treatment [Figure 1]. In previous decades, the indication for treatment was exclusively urinary retention, whereas today, LUTS secondary to BPH make up the largest patient population for whom treatment is offered.

**Table 1:** Injection therapy: intraprostatic routes, injectants, and clinical indications

Intraprostatic route	Injectants	Clinical indications
Transperineal injection	Pepsin and Pregl's solution 5-10% silver nitrate 10% sodium hydroxide lugol's solution 95% alcohol mixture of carbolic acid, glacial acetic acid, glycerin, and distilled water absolute ethanol	urinary retention treatment of BPH/LUTS
	various antiseptics and antimictobials	acute and chronic prostatitis
Transrectal injection	hypertonic saline liquid and gel absolute ethanol liquid and gel	treatment of BPH/LUTS
	various antiseptics and antimicrobials	acute and chronic prostatitis
Transurethral injection	aqueous mixtures of collagenase, hyaluronidase, triton X-100, and gentamicin absolute ethanol hypertonic saline botulinum toxin A (BONT-A)	treatment of BPH/LUTS
	absolute ethanol	pre-TURP intraprostatic injection for hemostasis

## ROUTES OF INJECTION

### Transperineal

Talwar and Pande's 1966 landmark study fueled the momentum for subsequent investigation of transperineal prostatic injection.[12] Using digital rectal guidance for prostatic needle placement, results from 188 consecutive cases revealed a 78% improvement of BPH symptoms using only injection therapy and a 2.6% recurrence rate over a three-year period. In the wake of this groundbreaking study, several publications attempted to popularize the procedure.[13,18] Having referenced Sir James Roberts and the use of transperineal injection (nearly 60 years earlier) for the treatment of BPH, it is of interest to note that Roberts had ostensibly trained Sharma in this injection procedure and, subsequently, Sharma trained Talwar and Pande.[12] The technique for the use of a spinal needle and digital rectal guidance for transperineal intraprostatic injection was illustrated in this notable literature. The transperineal route remains, historically, the most studied and evaluated injection approach.[12–14,16,18]

Increased practice of transperineal injection supported by multiple publications confirming its efficacy was overshadowed by the growing experience in the literature regarding transperineal treated patients complaining of post-injection perineal pain.[12,13,18] In 1970, Broughton and Smith revealed radiographic evidence of extraprostatic extravasation of a radiopaque injectant while performing transperineal injection in a patient.[18] With the obvious risk of extraprostatic injectant leakage and several subsequent publications reporting suboptimal success, intraprostatic injection using the transperineal route was largely deserted.

In 1988, the specter of transperineal intraprostatic injection was resurrected in the literature.[18] Seeking alternative treatments for localized prostate cancer, a group composed primarily of radiologists implemented a series of investigations using a canine model for transperineal intraprostatic injection of anhydrous ethanol (AE). Their study demonstrated the desired effect of focal interglandular prostatic necrosis in 71% of the transperineal injections. The procedure, however, was associated with complications of periurethral necrosis, external sphincter necrosis and bladder mucosal necrosis resulting from injectant leakage evidenced by ultrasound in a significant proportion of the study animals.

More recently, however, numerous publications have revisited the transperineal route of injection, with data supporting the efficacy of this therapy.[18–22] In each study, transrectal ultrasound (TRUS) was utilized for guidance and visualization during injection of small total-volume injectants. Real time TRUS may allow for definitive visualization of each injection, thus reducing the occurrence of intra-procedural injectant backflow.

Overall, the transperineal route of injection has demonstrated promise, but this approach is not without concern. Uncontrolled injectant leakage outside the extra-prostatic capsule by way of backflow along the needle tract may lead to significant injury to tissues other than the prostate.[12–16,18,23,24]

### Transrectal

There is a dearth of literature pertaining to transrectal routes for intraprostatic injection in the treatment of BPH. Our search revealed that the only publications specific to intraprostatic injection report on prostatic abscess puncture and/or the delivery of anti-infective agents for the treatment of infective prostatitis.[4,5] Three abstracts by Larson *et al*., have been referenced in the literature presenting separate transrectal intraprostatic injection experiences.[18,25] These reports involve the use of a needle guide with TRUS probe for visualization of intraprostatic needle placement on small numbers of patients with short-term follow-up. Transrectal injection may have potential pitfalls, the most serious of which is the risk of urethrorectal fistula formation, a complication previously reported with microwave thermotherapy.

### Transurethral

Technological advancements in endoscopy and cystoscopy have been the impetus for transurethral access to the prostate. In one of the earliest documented attempts to treat infectious prostatitis via transurethral injection, McCarthy, in 1935, injected a colloidal silver solution (also known as electrargol) via the “urethroscope” and “panendoscope” into each lateral lobe of the prostate. Overall, he treated 40 patients, 38 of which reported definite improvement and 16 discharged home as “clinically cured”.[26] In 1995, Mori *et al*., used absolute ethanol via transurethral injection prior to TURP for prevention of perioperative blood loss.[27] In 1996, Harmon *et al*., performed intraprostatic enzymatic tissue ablation via transurethral injection in canines.[28]

Transurethral injection was systematically revisited in humans by Goya *et al*.[29] In 1999, this group reported on an experience using a straight needle with cystoscopic injection into the lateral prostatic lobes to treat 10 patients with a three-month follow-up. Despite reports of promising results, no diminution of gland volume was seen in this cohort.

In a novel study published in 2006, Mutaguchi *et al*., used a straight needle with endoscopic guidance to analyze the efficacy of transurethral ethanol injection in 21 patients with persistent urinary retention secondary to prostatic obstruction. The group reported favorable outcomes in 17 patients. However, the size of the study and the relatively short follow-up warrant further investigation.[30]

In the last decade, there has also been discussion in the literature and at international meetings about the use of a curved needle with passive axial deflection allowing for deeper prostatic injection.[18,23,31,32] Although originally intended for *in situ* radio frequency (RF) ablation of prostate tissue, the hollow core configuration of the needle also allows for intraprostatic cystoscopic injection.[18,23,31] In 2002 we reported the only series of patients treated with this device and RF.[31] Unsatisfactory results with RF, however, led to the conception of substituting an injectable agent. Using a canine model, we then explored the feasibility of the device to inject an agent intraprostatically.[18,24,31] Subsequently, a small pilot study was conducted using anhydrous ethanol (AE) with promising results.[31] This translational research substantiates the theoretical advantage of transurethral injection over other injection routes - by preserving the integrity of the prostatic pseudo-capsule, the necrotic effects of AE can be limited to just the parenchyma of the prostate gland, thereby precluding extra-prostatic necrosis.[18,23,24,31,32]

In the following year, we performed a comparative analysis of transperineal versus transurethral intraprostatic injection using AE in 25 canines.[23] The transurethral approach was corroborated by the results as having less overall extraprostatic effects relative to the transperineal route when using AE as the injectant.

The curved needle device originally used for RF tissue ablation has since been redesigned with the removal of the RF coupler and the addition of a dιtente system for graduated needle deployment.[18,23,31,32] Pursuant to these modifications, several large international multicenter trials and two limited United States pilot trials were completed and reported promising results.[18,31] The use of this transurethral injection system intraprostatically constitutes the largest reported literature for the treatment of BPH. To date, more than 1500 patients worldwide have been treated. Most recently, a Phase I/II trial with a six-month follow-up examining transurethral injection of ethanol in 79 patients was published. The results have helped to further bolster the potential and promise of this device and technique for intraprostatic injection.[33]

## INJECTABLE AGENTS

Historically, a myriad of injectable agents have been employed in the treatment of BPH, including (but not limited to) acetic acid, carboxylic acid, enzymes, hyperosmotics, iodine, neurotoxins, a mixture of phenol, and now the most investigated and popularized injectable, 98% AE.[12–14,16,18,23,24,28,29,31,34] The consistent and desired effects for most intraprostatic injectables are the production of a host-mediated inflammatory response, localized coagulative necrosis and an overall diminution in gland volume.[12–14,16,18,23,24,28,29,31]

Despite the diversity of injectants considered, AE represents the agent most widely researched and clinically applied for *in situ* tissue ablation. Currently, AE by percutaneous injection is regarded as the standard of care for the intralesional treatment of hepatocellular carcinomas and parathyroid adenomas.[18] In the field of urology, past experiences and uses for AE include renal angioinfarction, injection subtrigonally for detrusor instability, and research quantifying intraoperative irrigant fluid absorption by patients during TURP.[18] Numerous past procedural experiences have confirmed the safety and efficacy of AE and maintain support for its espousal in other urologic applications such as intraprostatic injection.

In recent years, AE has eclipsed the international urology literature relating to its effective *in situ* prostatic tissue ablation properties.[18,23,24,29,31] Much of the previous research and experience regarding intraprostatic AE injection has been amassed outside of the United States (US) due to current US legislation governing new drug approval. Two small clinical pilot studies had been conducted in the US using AE injections,[18,31] which then stimulated the formal evaluation of Food and Drug Administration (FDA) approval as an investigational new drug (IND) for treatment of BPH (IND #61337). Earlier this year we published results from a Phase I/II IND trial evaluating the safety of transurethral ablation of the prostate (TEAP).[33] From a historical perspective, this was the first ever IND for a new injectable drug for the treatment of BPH.

Issues with regard to patient safety in several clinical experiences using transurethral AE intraprostatic injection have been addressed in the literature. To date, only two serious adverse events have been documented post-transurethral AE injection.[33] Investigations revealed that both incidents of patient morbidity were subsequent to electrocautery prostatic resection procedures for urinary retention months following the primary procedure.[35]

Ethanol has also been investigated in the gel form. In 2006 Larson *et al*., used a viscous solution of 97% denatured alcohol and a patented polymer for intraprostatic injection of 65 patients with BPH.[36] Theoretically, the viscous nature of the gel circumvents potential pitfalls experienced in studies using transperineal injection without TRUS guidance (extraprostatic extravasation, injectant backflow along the needle track). Although preliminary results have shown desirable improvements in patient outcome, additional clinical experience is warranted.

In 1996 Harmon *et al*., publicized the use of an innovative enzyme injectant using a canine model.[28] Transurethral intraprostatic injection of a combination of collagenase and hyaluronidase, two stromal-specific enzymes, the detergent Triton X-100, and the aminoglycoside gentamicin produced predictable and favorable histological responses in the canine prostate. To date, no subsequent follow-up human studies have been published.

Another injectable agent previously considered was hypertonic saline in liquid and gel form.[18] Despite its ablative properties in prostate tissue in murine and canine models, however, hypertonic saline, much like other intraprostatic injectants, has had limited published studies evaluating its clinical use.[18]

In the last five years the use of botulinum toxin A (BONT A), an exotoxin produced by the bacterium *Clostridium botulinum*, has become popular for a number of urologic indications.[18] Most recently, this was extended to include intraprostatic injection for the treatment of LUTS and BPH.[18] Injection of BONT-A in murine and canine prostate tissue has demonstrated a reduction in glandular volume. Currently proposed explanations for this phenomenon include neurotoxin-induced denervation atrophy,[18] as well as alteration of cellular dynamics through the induction of apoptosis (via blockage of ACh), inhibition of proliferation and down-regulation of α_1A_ -adrenergic receptors.[37] Preliminary results from several clinical experiences in over 150 patients, most with short-term follow-up, have shown promising results.[38] Use of intraprostatic injection of BONT-A, however, is currently not approved by the FDA. As such, additional laboratory research and clinical trials are required before widespread application.

The definitive injectable of choice will inevitably be based on the agent's availability, cost, safety and efficacy. To date, a comparative analysis of the previously reported injectable agents does not exist within the literature. In the US, any injectable agent exerting a biologic effect for the treatment of BPH requires a New Drug Application (NDA) to the FDA for its use as part of any medical procedure.

## INDICATIONS

Urinary retention, in the literature, was the sole indication for the treatment of BPH via intraprostatic injection until 30 years ago.[12–14,16,18] Increasing numbers of patients were studied in the ensuing years with symptomatic LUTS secondary to BPH.[18,23,24,31] Most recent reports primarily embrace this symptomatic patient population, as current treatment guidelines for the use of any minimally invasive BPH therapy specifically exclude individuals with urinary retention.[17] However, injection therapy, as a renaissance concept, once again has the attention of investigators seeking its use in the treatment of urinary retention.[30]

The severity of an individual patient's preexisting co-morbidities has been the common denominator among effectively all patient groups referenced for intraprostatic injection treatment. The application of this “new” procedure has been characterized as most appropriate for older patients with multiple co-morbidities designated as high-risk surgical candidates.[18,24,29,31]

## COMMENT

The last century has seen the acquisition of a vast wealth of knowledge with steadfast enthusiasm for this concept.[1,12–14,16,18,23,24,26–29,31,32] Enriched understandings of the pathophysiology of BPH, coupled with perpetual enhancements in technology, have augmented the evolution of the injection concept. Alas, many incomplete evaluations evident within the intraprostatic injection literature have limited the formation of solid scientific conclusions. Numerous anecdotal references to preclinical work in humans lack references and are devoid of crucial details such as study design.[13,16,18,27,29] Additionally, despite some literature on the diffusion properties of intraprostatic injectables using the canine model and small numbers of patients, this area of investigation is deficient and warrants a thorough evaluation.[2,18,23,29,39] As such, the canine prostate continues to provide the best animal model in the study of clinical treatments for BPH.[14,18,23,39] However, previous research has shown the existence of significant comparative differences in the canine and human prostate with regard to structure, physiology, and histology of the gland.[18] Accordingly, a systematic and comprehensive assessment of individual injectable diffusion properties in an appropriate number of human prostates will be necessary for a more elaborate understanding of this critical issue.

Also requisite to a heightened acumen of this topic are current investigative efforts directed towards deciphering the specific mechanisms of action of individual injectable agents. Presently, all injectables, save BONT-A, appear to induce varying degrees of intraprostatic coagulative necrosis orchestrated by a localized host immune response within the glandular capsule. Without documentation of any scientific comparison for prostatic application, the ideal injectable has yet to be described. Thus far, the practice of intraprostatic AE delivery via the transurethral approach is the frontrunner in the literature on the treatment of symptomatic BPH, yielding the largest number of publications and the largest total patient experience. At present, AE is definitively the agent of choice in other organ systems.

As the practice of medicine has evolved, so have the indications for the treatment of BPH undergone substantial changes. The en vogue medical treatments focus earlier on the natural progression of many diseases, including BPH, with the aim of hindering and possibly averting unwanted secondary complications in the ageing male.[1,17,18] This approach results in significant benefit in patient quality of life and a subsequent increase in numbers of patients seeking available treatments. Moreover, current trends of increasing elderly populations in cultures around the world lend much support to the continued investigation of a simple, safe and economical, minimally invasive alternative treatment for BPH.

## CONCLUSION

With a rich history in the urology literature, intraprostatic puncture and injection have seeded the accruing fund of knowledge referencing the use of intraprostatic injectables for the symptomatic treatment of BPH. Given the relative procedural ease and cost-effectiveness of intraprostatic injection, this technique has significant potential as a means of delivering a biologic or chemical agent for tissue ablation. This concept represents one of the oldest and most widely investigated minimally invasive treatment options for BPH.

Despite inchoate forms of early ideas, concepts, and the initial intent of the investigative forefathers in the field, the historically cyclical practice of intraprostatic injection has been revitalized for use in the treatment of BPH. It is our hope that continued interest and effort, including our own current research, may be fostered and petitioned for this important work to be advanced.
